# The Use of Non-physician Prescribed Medications in Patients Presenting to Two Emergency Departments in a Low/Middle-income Country

**DOI:** 10.5811/westjem.2022.2.54302

**Published:** 2022-06-17

**Authors:** Donna Venezia, Alexandra Cabble, Diane Lum, Kruy Lim, Adam J. Singer

**Affiliations:** * Stony Brook University Medical Center, Department of Emergency Medicine, Stony Brook, New York; † Stony Brook University Medical Center, Stony Brook, New York; ‡ NYU Langone Health, New York, New York; § Sihanouk Hospital Center of Hope, Phnom Penh, Cambodia

## Abstract

**Introduction:**

With few trained healthcare practitioners and limited personal finances, many patients in low/middle income countries purchase prescription medications from non-physician prescribers (NPP). This study documents various aspects of this practice, including patterns of prescribing, and the patient’s understanding of medication risks.

**Methods:**

From January to April 2017, 479 patients entering two hospitals in Phnom Penh, Cambodia, were surveyed. Demographics, medications, types of NPP who provided the medication, patients history and physicians’ chart data were documented. Information, including symptoms when the medication was purchased, possible side effects, hospital presenting symptoms, etc, was recorded. The patient’s understanding of medication allergies and risk of serious side effects was also documented.

**Results:**

Of the 467 patients included, more than half (59%), reported buying medications from NPPs within the two weeks before presenting to the hospital. Nearly half of those patients, (42%), could not identify any of their medications. Of those 159 patients who could identify at least one drug, 79% bought at least one medication that would require a prescription in the United States. Only 8% of patients were aware that medications could cause serious harm. Twenty-three percent of the known medications were oral or injectable corticosteroids, and 56% of steroid users, typically chronic users, had evidence of possible side effects.

**Conclusion:**

Many patients in one low/middle income country received prescription medications from various NPPs with little information concerning these medications. Efforts to educate the public about their medications and the potential risks of medications are needed.

## INTRODUCTION

### Background

Cambodia is a rapidly developing country in Southeast Asia that suffered a decimation of its doctors and medical staff during the Pol Pot regime in the 1970–90s. According to 2014 World Bank data, 40% of the countries around the world have fewer than one doctor/1000 people, with Cambodia having only 0.2 doctors/1000.^1^,^2^ In particular, Cambodia’s rural areas are often without any physicians. Non-physician prescribers (NPP) frequently fill the void in those regions, though many may have no formal medical training. Some NPPs focus primarily on selling medication, providing guidance to patients as to which medication to buy based on their symptoms. Other NPPs may open private practices and, in addition to providing advice after clinical evaluation, may sell medication. Some of these NPPs may be trained nurses, though unlikely trained as nurse practitioners. Other practitioners, however, may have only been trained as a nurse’s aide observing physicians in a hospital setting or, in earlier times, at a refugee camp, before opening their own practice. Small villages may have governmental health centers, some staffed with trained mid-level healthcare personnel, others with staff who have uncertain training.

**Figure 1:**
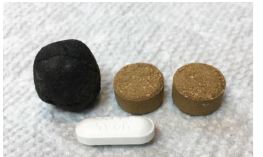
Examples of “Khmer traditional medication” with possible admixture of prescription medication. (Acetaminophen caplet for size comparison.)

Licensed pharmacists may also be limited in these regions. Even where pharmacies exist, the staff behind the counter providing prescription advice may have had no pharmaceutical training but may simply be the pharmacist’s relative, or other non-medically trained staff. Retail shops can also sell prescription medications, with the store clerk providing medication guidance. Finally, medications and medical advice may come from village healers providing traditional Khmer medication.^3^ While traditional medication is typically herbal, a number of Cambodian doctors believe prescription medication may be added into some of the herbal mixtures, creating a potential source of unknown prescription medication. Literature describing this practice is difficult to find. The Ministry of Health has reportedly banned this type of mixed “traditional” medicine, but hospital staff indicate it is still sold **(**Figure 1).

### Importance

In the United States, where prescription medication is only provided by licensed healthcare workers, medication errors account for a significant proportion of overall medical errors.^4^,^5^ However, in many, if not most, parts of the world, prescription medication can be bought by patients without a doctor’s prescription or consultation with an appropriately licensed practitioner.^6^–^26^ This may be because doctors are unavailable, lack of trust in the medical establishment, or patients simply can’t afford both a doctor’s consultation and the recommended medications. While information regarding the dispensing of antibiotics without prescriptions by pharmacists can be found in the literature, limited information is available concerning the scope and details of all types of non-physician prescribing.^27^–^32^ Additionally, many global healthcare volunteers coming from countries with highly regulated prescription practices may initially be unaware of the various types of informal prescribing practices present in developing regions.

*Population Health Research Capsule*

What do we already know about this issue?

*Antibiotics are frequently bought in low/middle income countries without an approved healthcare clinician’s consultation, yet little has been documented concerning other medications.*

What was the research question?

*What is the scope of all types of prescription medication usage without consultation? Do patients understand medication risks?*

What was the major finding of the study?

*More than half of 467 patients (59%), reported buying medications from non-physicians and nearly half (42%), could not identify them. Three-quarters required prescriptions in the US. Only 8% were aware they could cause serious harm.*

How does this improve population health?

*This study highlights the need for patient education concerning medication usage and risks in one low/middle income country.*

### Goals of this Study

This study has two goals. First, the study describes the scope and details of the prescribing practices of NPPs in one low/middle income country, including patterns of prescribing for the most commonly used medications and possible side effects from these medications. Second, the study attempts to characterize the patients’ understanding of medication adverse effects and risks.

## METHODS

### Study Design and Setting

This prospective, observational study was conducted between January and April 2017 at two sites: (1) Sihanouk Hospital Center of Hope (SHCH) and (2) Hope Worldwide Community Health Center (CMC)], in Phnom Penh, Cambodia, after institutional review board approval by the participating medical centers and the Cambodian Ministry of Health. The two sites included emergency departments with approximately 90 patient visits/day, one for paying patients and one for indigent patients. Both sites are staffed by the same group of physicians. The two hospitals primarily see adult patients with various medical problems. Pediatric, gynecology/obstetric, psychiatric and major trauma patients are rarely seen in the EDs because most patients self-refer to other hospitals. Known human immunodeficiency virus (HIV) patients were seen by infectious disease physicians separately.

### Selection of Participants

**Figure 2:**
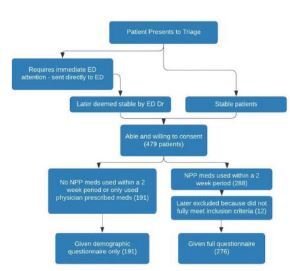
Enrollment criteria for stable patients in Cambodia studied for identification and knowledge of their current medications. *ED*, emergency department; *NPP*, non-physician prescriber.

A convenience sample of stable, consenting patients visiting one of the two study sites was eligible for enrollment after triage by a nurse (Figure 2). Two trained, bilingual Cambodian nurse research assistants (RA) assessed whether patients met inclusion criteria and then verbally administered the survey to all patients. All patients were eligible except those requiring immediate medical care. However, those not initially eligible could become eligible for enrollment after their condition had stabilized. All patients gave written or thumbprint informed consent.

### Intervention and Measurements

A 34-item questionnaire (Appendix A) was verbally administered by a trained RA to those using medication provided by NPPs within two weeks before presentation. This questionnaire covered medication name, dose/duration, expiration dates, prescriber, reason patient requested treatment, and the response to treatment; ie, did they improve or develop new symptoms/possible side effects after starting the medication. Patients were asked whether they requested medical advice from the prescriber and if the prescriber asked questions, such as other symptoms, past medical history, other medications, allergies, or pregnancy. Also noted were prescriber instructions (verbal only or written), prescriber information concerning adverse effects, and the patient’s understanding of the term “medication allergy” and serious side effects.

After physician evaluation, information was obtained from the medical chart including discharge diagnosis, past history, and ancillary tests. Medication side effects were included as “possible” side effects, if a new symptom had started after the medication had been used and this symptom is a known side effect of that drug. Specific medication side effects were not asked by the RAs, but once a patient reported new symptoms, the RAs and the authors (some of whom were present during the first month of data collection) were encouraged to ask further details to help clarify the likelihood the symptom was related to the medication. For instance, one patient who self-prescribed chronic steroids for no known reason, brought in recent radiographs demonstrating progressive bilateral femoral head necrosis. He was further questioned concerning other possible known risk factors associated with the condition (none were noted), and so was included as a “possible” medication side effect. Patients using both non-steroidal anti-inflammatory drugs (NSAID) and steroids for chronic pain frequently complained of post-use gastric irritation, and some were noted to have iron deficiency anemia. While the NSAIDs alone could have caused the symptoms or the patient may have had undiagnosed anemia even before the start of the medication, this would still have been included as a “possible” steroid side effect since the medication combination is known to increase the risk of gastric irritation and bleeding.

### Outcomes

First, the study documents various aspects of NPP prescribing practice. This also included evaluating prescribing patterns for the most commonly used medications (determined after the study’s completion) and noting any possible medication side effects reported by patients for these medications. Secondly, the study evaluates the patient’s understanding of medication risks.

### Data Analysis

Descriptive statistics were used to summarize the data. Categorical data were summarized as counts and percentages frequency of occurrence. Continuous data were summarized as means and standard deviations. Because this was not a hypothesis-testing study, no formal sample size calculation was performed. The study was conducted over a four-month period during which funding and research personnel were available.

## RESULTS

### Characteristics of Study Subjects

During the study period, 479 patients were enrolled. Twelve were excluded, either because their recorded medications were used before the two-week study cutoff or a physician potentially prescribed their medications. Of the remaining 467 patients, 276 (59%) patients reported buying medications from various NPPs within the two weeks before presenting to the hospital. Mean age was 53 years (range 15 – 85) and 68% were female. Patients travelled an average of 2–2.5 hours from home to hospital, 72 % required assistance to pay for services, and 70% were able to read at an elementary level or higher. There were 191 (41%) patients who did not use any medications or only physician-prescribed medication in the two weeks before presentation.

### Main Results – Prescribing Practices

Of the 159 patients who knew the name (or brought in the labels) of at least one medication, 79% purchased at least one medication that would require a prescription in the US. The 176 known prescription medications commonly fell into several categories: antihypertensives 44 (25%); oral/intravenous steroids 40 (23%); oral antibiotics 35 (20%), oral antihyperglycemics 19 (11%); and sedatives/narcotics 15 (9%). Twenty-three (13%) other types of medications were also documented. This data does not include those who used unknown injectables (n=53), most of which would require a prescription in the US. It also does not include the “Khmer traditional medication” (n=32) that RAs believed, based on the patient’s description, might be mixed with prescription medications. Patients who described taking purely herbal Khmer traditional medications were not included.

The most common “prescribing” source was a “retail” source (45%), either a store salesperson or an individual medication seller who helped guide the patient in buying medication. **The **table lists other prescribing sources. A small number of patients were unable to distinguish whether their prescriber was a doctor or another type of practitioner. Others could not distinguish a store clerk from a pharmacist.

**Figure 3:**
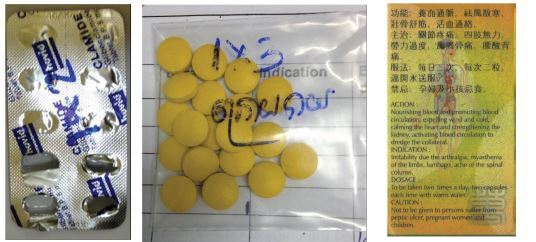
Examples of “written instructions.” Many patients received medication in a plastic bag with simple dosing directions written with a marker on a plastic bag, but usually without the drug name. Occasionally, blister packs were given (name typically in English), with dosing instructions written on the blister pack. Less frequently, a pre-packaged bottle/box was given with instructions typically written in English, French, Thai, or Chinese.

Few NPPs asked patients for additional medical information before prescribing. For example, only 17 patients (6%) reported being asked about allergies, and most of these patients already had a history of allergy and likely informed the prescriber themselves. Only 13 sellers (5%) provided any information about the potential adverse effects of the medications; however, most sellers did provide dosing instruction. One hundred eighty-eight patients (68%) received only verbal instructions, and 56 patients (20%) received written instructions. Thirty-one patients (11%) did not request any help when purchasing the medication. Figure 3 provides common examples of “written instructions.”

### Patient Understanding of Medication Risks

Of the responding 273 patients, (90%) did not understand the term “medication allergy.” The 10% that did typically had suffered a medication reaction in the past. Twenty-six NPP medication users (9%) stated they had an allergy, describing symptoms that could be consistent with a possible allergy, but many were not sure which past medication caused the reaction. Only 23 patients (8%) were aware that medications could cause serious reactions (again, usually those with past drug reactions).

### Prescribing Patterns and Reported Side Effects

While the inclusion criteria required patients to have purchased NPP medication within the prior two weeks before presentation, many medication treatments had actually begun months or years prior, particularly anti-diabetic medication, anti-hypertensives, and steroids.

Corticosteroids were a common class of medication for which patients reported new symptoms after starting the medication. While only 40 patients either brought in or knew the exact name of the steroid they were using, an additional 21 patients either (1) knew they were taking a steroid but didn’t know the specific name, (2) had documented physical evidence they were likely taking a steroid (ie, developed “moon face” along with other common side effects, such as “fragile” skin after taking the unknown drug chronically), or (3) had a physician’s diagnosis stating that the patient was taking chronic steroids. Three excluded patients took NPP steroids before the two-week cutoff and had possible side effects affecting the present diagnosis, ie, “moon face” and possible symptoms of adrenal insufficiency. If these additional patients were included, then 64 (13%) of all 479 patients or 64 (22%) of 288 NPP users, including excluded, had chart evidence of steroid usage.

Though formal confirmation of side effects was not possible given the study design, 36 (56%) patients using steroids reported a number of possible side effects, including bilateral femoral head necrosis, poorly controlled or new onset diabetes/hypertension (some showing improvement after stopping the steroid), new epigastric pain with evidence of iron deficiency anemia, (frequently in those also taking an NSAID), skin changes (“fragile” skin, bruising), weight gain, and edema. Additionally, a few patients demonstrated possible, but unconfirmed, signs of adrenal insufficiency after they recently stopped using chronic steroids.

Pain (“total body pain,” joint, back, or muscle pain, et.) was the most common hospital presenting complaint for NPP users (40%). It was also the most common reason steroids were prescribed (desired weight gain, respiratory issues, toothache, and rashes were less common reasons). While patients were typically prescribed steroids for only a few days, many with long-term pain returned for more, with 44 (69%) having chart evidence of chronic usage. “Moon face” was a hospital presenting complaint for seven (3%) of our triaged patients, and this side effect was noted by an additional 17 patients.

An unknown intramuscular or intravenous injection was the most common “prescription” therapy received in the two weeks before presentation by 53 patients (24% of NPP users if added to the known prescriptions medication). Eighty-nine NPP users (32%) also admitted to past injection therapy. Only one patient received a subcutaneous injection, possibly insulin, (which may not have necessarily required a prescription in the US.) Only three patients knew the names of their injectable medications (two steroids, vitamin C, and meloxicam). Patients generally bought the medication from one source and then called “someone who knows how to inject” to their home to administer it. Occasionally, a retail seller performed the injection, and a few injections were intra-articular.

The NPPs frequently used glucometers, lab results brought in by patients, and/or blood pressure cuffs to sell antidiabetic and antihypertensive medications, often appropriately when compared to hospital results. However, many patients reported not receiving any information concerning diabetic medication side effects. Of the two diabetic patients who suffered hypoglycemic reactions, neither was aware that eating less might result in hypoglycemia. Of the 30 patients who knew they were diabetic, 26 were taking an anti-diabetic medication, although seven did not know the exact name. Of these diabetic patients, 18 (69%) presented to the hospital with hemoglobin A1C >7 % (for chronic diabetes mellitus) or if newly diagnosed (less than three months) and treated by the NPP, with a serum glucose greater than 200 milligrams per deciliter.

Of the 50 patients who knew they were taking an antihypertensive medication, 17 (34%) reported taking the medications only “when blood pressure was elevated,” and 19 (38%) stated they used it “on and off” for symptoms such as “headache and neck tension.” One patient thought a short course of medication would cure hypertension.

Antibiotics were frequently prescribed for non-infectious conditions. For example, 16 patients (42%) were prescribed an antibiotic for chronic knee pain. Additionally, many of those prescribed an antibiotic for an infectious etiology would not have benefited because the disease ultimately diagnosed at the hospital required a different or more complex form of treatment, ie, septic arthritis, tuberculosis, melioidosis, etc.

Examples of specific medication issues in selected patients can be found in Appendix B.

## DISCUSSION

Though this study has inherent limitations, it does shed light on several issues concerning non-physician prescribed treatment. Many Cambodians lack easy access to physicians, but more than half were able to obtain NPP-prescription medication before presentation to the hospitals. While many non-physician “prescribers” frequently left patients with little information concerning the treatment they had purchased, it is important to note that this study did not compare this practice to physician prescribing; so it cannot be assumed that treatment would have been any different.

We did not specifically count the number of patients given medications without written names; however, many medications were dispensed in this manner (ie, placed in a plastic bag without a name). Without drug names written in the patient’s native language or information concerning adverse effects, it becomes difficult for patients to participate in their own healthcare. Many patients are not able to choose commonly used over-the-counter medication on their own simply because they can’t read the labels typically written in languages different from their own. It is difficult to identify adverse drug events and even more difficult to prevent future episodes when names are unknown. Failed therapies from the past cannot easily be identified, since once a drug is finished, there is rarely any documentation of the medication prescribed. Verbal information alone may have sufficed in the past when many could not read, but, in our population, more than two thirds of patients were able to read at a basic elementary level and many non-readers have younger family members who could read for them. Written medication information should be provided to all, in their own language, and it is important to emphasize that such information must be saved and brought to subsequent healthcare staff.

Our survey questioned patients about potential medication side effects; however, it would be difficult, considering the study design, to confirm with absolute certainty that the symptoms patients experienced after usage were necessarily caused by the medication. However, a number of patients did report a variety of new symptoms particularly related to chronic steroid usage. While Cambodian physicians at the hospitals are aware that patients may be using chronic steroids and specifically search for symptoms/signs, most patients were completely unaware of steroid risks since this was not communicated to the patient when the medication was sold.

With strong anti-inflammatory effects and rapid improvement of many varied inflammatory symptoms, a number of patients returned for more, some believing, as one patient stated, “steroids cure everything.” This belief may be the reason why steroids might be added to traditional Khmer herbal medicines. While we did not conduct chemical analysis on any of the patient’s “traditional medications,” there is evidence suggesting the practice of mixing prescription medications in herbal concoctions may be more common than recognized. One patient bought an herbal medication from Malaysia that has been banned in the US after a Food and Drug Administration analysis revealed the medication was not purely herbal, but contained steroids and cyproheptadine.^35^ Steroid additives to herbal medication have been reported by others in the Southeast Asian region and have resulted in unexpected secondary adrenal insufficiency during stress such as during surgery.^36^–^38^ Two patients who reported *only* using Khmer traditional medication presented with “moon face” after taking the “traditional” medicine chronically, making it highly likely that a corticosteroid was indeed an additive to those Khmer traditional pills. Chinese herbal medications, also used by Cambodians, may also be mixed with steroids, but our study did not independently confirm this.

Knowledge (or lack of knowledge) of chronic steroid use affected the differential diagnosis and management of certain patients with infections. One patient with a long history of steroid use presented with clinical signs of sepsis, progressive fever, shortness of breath, and weakness, and died soon after admission. Although a specific infectious diagnosis was not made, her management and differential were influenced by the knowledge of her chronic steroid usage. Her treatment included stress dose steroids and treatment for possible strongyloidiasis, a common asymptomatic local parasitic infection caused by *Strongyloides* that is known to disseminate when using steroids.^39^–^40^

Our present study was also not designed to pick up risks from the common practice of injecting medications; however, there has been past evidence of risk. A few years ago, the Ministry of Health actively discouraged this practice after a NPP was discovered to have infected multiple patients with HIV by reusing the same non-sterile needle to inject his patients.^41^ Despite the publicity, local doctors state patients still frequently request injections. Injections were found to be the most common type of prescribed medication in this study, yet virtually no one knew the medication that was administered. This is an area that would likely benefit from further studies.

While this study may not be able to generalize the specific findings to other regions of the world, it is likely that many of these same issues can be found in other locales . For example, in addition to issues with steroids/herbal mixtures found in other Southeast Asia locales, a similar problem with injectables occurred recently in India when a NPP also reused needles causing multiple HIV infections.^42^

This study cannot determine the true risk/benefit ratio of NPPs advising patients on medications, because those patients that improved would not likely come to a hospital. A study at the source of the sale might better characterize the overall risk/benefits of the NPP practice. Additionally, a physician comparison group would further aid in evaluating the risk/benefit.

Non-physicican providers may provide a great benefit to patients, particularly those with chronic diseases, such as hypertension and diabetes, who do not have easy access to a doctor. Though two thirds of our NPP-treated diabetic patients presented with subsequent evidence of poor glucose control, one third demonstrated good control and this may not have been possible without local NPPs. Additionally, having a physician rather than a NPP provide treatment may not necessarily result in better glucose or blood pressure control since poor control may simply reflect the patient’s inability to pay for long-term treatment, rather than who advised them. However, one patient, diagnosed with diabetes by a physician, informed the RA that her doctor was insistent she take her medications daily, so she did. She was later diagnosed with hypertension by a NPP but did not receive similar instructions, so only took that medication “on and off.” Simply diagnosing a disease and providing a pill may not be enough. The NPPs should have an understanding of the disease for which they are providing medications in order to correctly advise the patient.

Providing more trained medical workers, such as physicians and pharmacists, to help patients gain a greater insight into the medications they have purchased may take years. Stronger governmental oversight or intervention of medication prescribing may take time, too. However, patient education can begin now. Nurse educators could offer general medication sessions including information about medication allergies, common adverse reactions, and information about long-term disease management while patients and families wait at local clinics and hospitals. Basic pre-printed medication information may be useful for both NPPs and patients.

## LIMITATIONS

The majority of the data in this convenience sampling of patients was based on patient self-report and may suffer from recall bias. Other study limitations include misinterpretations due to language and cultural differences, small sample sizes, accuracy of medical chart data, and the subjective nature of data analysis. Moreover, selection bias likely occurred as many of the sickest patients were not included in the study. Conversely, many patients prescribed medications by NPPs may never present to a healthcare facility because they improved. This study also lacks a physician comparison group; so the findings may not be representative of NPPs alone. Finally, the study may not be representative of other countries or regions in Cambodia as it was conducted at only two emergency departments in the capitol of Cambodia.

## CONCLUSION

Many patients in one low/middle income country received prescription medications from various NPPs with little information concerning these medications and their possible side effects. Education is not only essential, but key to decreasing the risk of iatrogenic disease and helping patients become active participants in their own healthcare.

## Supplementary Information





## Figures and Tables

**Table:** Source of medications.

Who prescribed medication	Number (%), total sources (N = 344)*
Retail store clerk or individual medication seller	156 (45)
Patient uncertain whether store clerk or pharmacist **	47 (14)
Non-physician healthcare worker at village governmental clinic	46 (13)
Non-physician healthcare practitioner (may or may not have medical training)	25 (7)
Pharmacist	20 (6)
Patient uncertain whether physician or non-physician practitioner ***	17 (5)
Friend/relative	17 (5)
Self	16 (5)

* Some patients (N = 65) used multiple sources when purchasing medication.

** Pharmacies may hire sellers who advise patients but may not be pharmacists.

*** Research assistants were allowed to include “uncertain” practitioners if, based on location (ie, rural) and other factors (ie, signage on front door), it was determined the chance of being a physician was highly unlikely.
