# Supplementary material for: The Use of Non-physician Prescribed Medications in Patients Presenting to Two Emergency Departments in a Low/Middle-income Country
Source: West J Emerg Med. 2022 Jun 17;23(4):540–7. doi: 10.5811/westjem.2022.2.54302 (PMC9391020; doi:10.5811/westjem.2022.2.54302)
Supplement: Supplementary file 1 [file wjem-23-540-s001.docx]

**Patient Data Collection Form–History Form A**

Research Assistant Name: __________________________________

(Data collected on all patients presenting to triage at the medical facility, unless unstable, then data collected only after physician permission.)

Informed consent given by patient/accompanying relative, if patient unable?

___Yes ____No

**IF NO 🡺 DATA COLLECTION STOPS FOR THIS PATIENT.**

If yes, continue with the form below.

Patient ID: __________________________________ Age: ________ Sex: ________

Province: ___________________

Transportation Time to SHCH/CMC __________minutes

MD/clinic within 30 minutes of home:

____Yes ___ No

Pharmacy/Drug Seller within 30 minutes of home:

____Yes ____No

Economic status: ____ Paying ____ Non-paying

Educational level: __________________

Able to read and write:

___ Yes ____ No

Chief complaint at this time: ______________________________________________________________________ ______________________________________________________________________ ______________________________________________________________________

Has the patient taken any NPP medication in the past 2 weeks?

___ Yes ___ No

If yes, continue with form B.

**IF NO 🡺 DATA COLLECTION STOPS FOR THIS PATIENT.**

**Data Collection Form–NPP Medication History Form B**

Describe reasons or symptoms that caused the patient to purchase/use NPP medications?___________________________________________________________________ ______________________________________________________________________________ ______________________________________________________________________________ ______________________________________________________________________________

The medication(s) was/were obtained from: Check all that apply

____a pharmacy/drug retail store

____friend or relative

____already had medication at home

____other ______________________________

Can patient/relative name the medications she/he has been taking?

___Yes ___No

How many different NPP medicines have been prescribed/used in the past 2 weeks? _____

Does patient/relative have the medications with them?

____Yes ____No

Can a friend or relative obtain the medication/labels or text/email a photo?

___Yes ___No

If purchased at pharmacy/drug seller, did patient give symptoms to a pharmacist and/or ask for help deciding on recipe of medications needed?

____Yes ____No

Who purchased the drugs at the pharmacy/drug seller?

____Patient _____Relative/Friend

Did the pharmacist/drug seller ask any other questions before prescribing?

____Yes ___No

If, yes, check all that apply:

___Allergy history

___other symptoms

___if childbearing age female, pregnancy possibility

___other meds or medical history

Were any instructions given to the patient as to how to take the medications?

____Yes ____No

If yes, were they written or verbal?

______Written _____Verbal

Were any NPP injection medicines prescribed?

____Yes ____No

Who administered the injectable medicine?

____ Patient ____ Pharmacist____ Relative/Friend

Has patient ever received or administered NPP injectable medicine before?

____Yes ____No

Were any instructions given concerning side effects of the medications?

____Yes ____No

Does the patient feel the NPP medicines improved or cured the problem that caused him/her to use the medicines?

___Yes ___No

If yes, is the patient at the medical center now for an entirely different reason?

____Yes ___No

Has the patient had any new symptoms after starting the NPP medications?

____Yes ____No

Describe.____________________________________________________________________________________________________________________________________

Documented medication record:

| **Name** | **Dose** | **Frequency** | **Duration** | **Indication** | **Expiration date** |
| --- | --- | --- | --- | --- | --- |
|  |  |  |  |  |  |
|  |  |  |  |  |  |
|  |  |  |  |  |  |
|  |  |  |  |  |  |
|  |  |  |  |  |  |
|  |  |  |  |  |  |
|  |  |  |  |  |  |
|  |  |  |  |  |  |

Does the patient understand "medication allergy"?

____Yes ____No

Is the patient aware medications can sometimes cause serious reactions?

____Yes ____No

Does the patient have known allergies?

____Yes ____No

Describe ______________________________________________________________________ ______________________________________________________________________

**Old Records and Present Visit–Data Collection Form C**

Has the patient been seen at the medical center before?

____Yes ____No

Data obtained from old records and present visit

____Yes

Data obtained only from present visit

____Yes

Chronic conditions: *Please check all that apply*

| CHF | Lupus | HIV/AIDS | Asthma |
| --- | --- | --- | --- |
| Hypertension | Anemia | GERD |  |
| Congenital heart disease | Rheumatoid arthritis | Chronic kidney disease |  |
| Thyroid disease | Epilepsy | Depression |  |
| Diabetes | Liver disease | Anxiety |  |

**Medication History**

*Previously prescribed medications from clinic*

| **Name** | **Dose** | **Frequency** | **Duration** | **Indication** |
| --- | --- | --- | --- | --- |
|  |  |  |  |  |
|  |  |  |  |  |
|  |  |  |  |  |
|  |  |  |  |  |
|  |  |  |  |  |
|  |  |  |  |  |
|  |  |  |  |  |

Vitals upon presentation:

HR: _______ BP: ________ O2 sat: _____ Temp: _______ RR: _______

Significant lab values: Creatinine_________ Liver panel________ H/H________ Glu______ other__________________________________________________________________

Admitting or discharge diagnosis for this visit: _____________________________________ _____________________________________________________
