# Supplementary material for: The Use of Non-physician Prescribed Medications in Patients Presenting to Two Emergency Departments in a Low/Middle-income Country
Source: West J Emerg Med. 2022 Jun 17;23(4):540–7. doi: 10.5811/westjem.2022.2.54302 (PMC9391020; doi:10.5811/westjem.2022.2.54302)
Supplement: Supplementary file 2 [file wjem-23-540-s002.docx]

**Appendix B**

Appendix B includes examples of 15 surveyed patients with author commentary. The non-physicians who prescribed the medications, the initial symptoms prompting the patient to request the medications, and any pertinent patient history, exam and/or labs, along with the hospital Emergency department presenting symptoms and the final diagnosis are included.

NPP 001 Age: 48 Sex: M

| **Name** | **Who prescribed** | **Dose** | **Frequency** | **Duration** | **Indication** | **Exp. date** |
| --- | --- | --- | --- | --- | --- | --- |
| **Paracetamol** | **Non Dr/Pharmacy** | **500 mg** | **tid** | **2d** | **fever** | **8-17** |
| **Ibuprofen** | **Non Dr/Pharmacy** | **200 mg** | **tid** | **2d** | **N/A** | **8-17** |
| **Doxycycline** | **Non Dr/Pharmacy** | **100 mg** | **bid** | **2d** | **N/A** | **2019** |
| **Vitamin C** | **Non Dr/Pharmacy** | **500** | **bid** | **2d** | **N/A** | **Not visible** |
| **Glibenclamide** | **Non Dr/Pharmacy** | **5 mg** | **bid** | **2d** | **N/A** | **Not visible** |
| **Azithromycin** | **Non Dr/Pharmacy** | **500 mg** | **bid** | **2d** | **N/A** | **Not visible** |
| **Fenoget (Fenofibrate)** | **Non Dr/Pharmacy** | **67 mg** | **qd** | **2d** | **N/A** | **5-17** |
| **Albendazole** | **Non Dr/Pharmacy** | **400 mg** | **qd** | **1d** | **N/A** | **7-18** |
| **Vitamin B12** | **Non Dr/Pharmacy** | **10 mg** | **bid** | **2d** | **N/A** | **9-18** |
| **Anticough (Thai med)** | **Non Dr/Pharmacy** | **N/A** | **N/A** | **2d** | **N/A** | **12-18** |
| **Sorbitol** | **Non Dr/Pharmacy** | **5 mg** | **N/A** | **N/A** | **N/A** | **11-18** |

Initial symptoms: Chill, fever, headache, low back pain

Hospital presenting symptoms: patient brought by family because he awoke confused/unresponsive since

last evening; had cough, weight loss for 1 month, fever for 1 week

Final Diagnosis: 1. Unresponsive secondary to hypoglycemia 2. Probable miliary TB [1st degree relative

with active TB and highly suggestive CXR], 3. History of ETOH use and liver disease.

Admitted SHCH.

Additional Info: BP: 144/87, HR: 102, O2sat: 98 T: 36.7 (axillary) RR: N/A (grossly normal); WBC: 9.4,

SGOT/SGPT: 41/76, Glucose: 1.5 mmol/L (nl: 4.1-6.1), HbA1C: 4.96 (nl 4.8 -5.9); CXR done. Patient went

to pharmacy 2 days before hospital presentation and was told to first get blood tests in nearby lab then see

the NPP associated with pharmacy, who ordered the meds. In ED patient awoke after one ampule of D50.

Initial ED diagnosis as to cause of hypoglycemia was uncertain. Meds were brought in by family next day. TB

meds started after consultation with Infectious Disease committee; transferred to TB hospital.

Authors’ Note: Although patient had no history or symptoms (i.e. blurry vision, urinary frequency) of DM and

had normal HbA1C: 4.9 in hospital, he was prescribed antidiabetic medication by an NPP/pharmacy just

a few days earlier, likely causing the hypoglycemic coma, (possibly a non-fasting lab test came back with an

elevated glucose). The patient stated pharmacy never gave DM teaching or information on adverse reactions,

so missed meals. Because patient had no reason to be on antidiabetic meds (no history of DM/symptoms),

doctors at hospital initially felt hypoglycemia possibly related to sepsis or ETOH use and did not suspect the

medication he was just prescribed until family brought them in the next day. The diagnosis of likely military

TB was missed and antibiotics were started that would likely not benefit this patient.

Abbreviations: Non-physician prescriber (NPP), Male (M), Expiration (Exp), Blood pressure (BP), heart rate

(HR), Oxygen saturation (O2sat), Temperature (T), Respiratory rate (RR), milligram (mg), every day (qd),

Twice per day (bid), Three times per day, (tid), Day (d), Not available (N/A), Chest X-ray (CXR), Ethanol (ETOH),

Sihanouk Hospital Center of Hope (SHCH), White blood cell count (WBC), SGOT/SGPT (liver transaminases),

Hemoglobin A1C (HbA1C), millimole per liter (mmol/L), Normal (nl), Emergency Department (ED), Dextrose

(D50), history (hx), Diabetes mellitus (DM), Tuberculosis (TB)

NPP 009 Age: 60 Sex: F

| **Name** | **Who Prescribed** | **Dose** | **Frequency** | **Duration** | **Indication** | **Exp. date** |
| --- | --- | --- | --- | --- | --- | --- |
| **Amlodipine** | **(Pharmacy/retail)**  **(Friend/patient)** | **5 mg** | **qd** | **1 m** | **hypertension** | **11/19** |
| **Unknown injection** | **Non-Dr health care worker** | **N/A** | **once** | **once** | **energy** | **N/A** |
| **Unknown injection** | **Non-Dr health care worker** | **N/A** | **once** | **once** | **Nerve painful** | **N/A** |

Initial symptoms: Headache, body painful, chest tightness for 2 days (Heard from a friend at work that

Amlodipine was good for hypertension, so she had her BP checked at a hospital (BP 180) and then went to

buy the med by herself; has been taking it for one month).

Hospital presenting symptoms: right sided weakness for 2 days (First called the local health clinic one day

after symptoms and a non-Dr came to her house and injected her with 2 meds for “painful nerve” and

“energy”).

Final Diagnosis: 1. Stroke, right-sided weakness 2. Hypertension for 10 years 3. DM II for 10 years

Additional info: BP: 149/75 HR: 85 RR: 19 Temp: 36.9 O2 sat: 98; Creatinine: 65 mmol/L,

Glucose: 376mg/dl, HbA1C: 10.13, SGOT/SGPT: 20/12, H/H: 12/36, CXR done. Has received NPP

injections in the past. PMH: DM, HTN

Authors’ Note: Uncertain who initially diagnosed patient with hypertension and DM 10 years ago, but

patient does not appear to understand the necessity of chronic therapy. Had consistent treatment

been emphasized 10 years ago perhaps the risk of stroke may have been lessened. After the stroke, the

NPP healthcare worker came to her home and administered two injections for “energy” and “painful

nerve” although uncertain how these might benefit patient. However, evaluation and treatment of her

other known condition, diabetes, was not initiated at that visit.

Abbreviations: Female (F), Month (m), Hemoglobin/hematocrit (H/H), Past medical history (PMH),

Hypertension (HTN)

NPP 049 Age: 61 Sex: F Excluded because stopped meds 3 weeks ago

| **Name** | **Who Prescribed** | **Dose** | **Frequency** | **Duration** | **Indication** | **Exp. date** |
| --- | --- | --- | --- | --- | --- | --- |
| **Dexamethasone** | **retail** | **0.5 mg** | **qd** | **N/A** | **inflammation** | **N/A** |
| **Unknown (round)** | **retail** | **N/A** | **qd** | **N/A** | **N/A** | **N/A** |
| **Unknown (oblong)** | **retail** | **N/A** | **qd** | **N/A** | **N/A** | **N/A** |
| **Penicillin Injection** | **Retail/health care worker** | **500,000 U** | **qd** | **N/A** | **antibiotic** | **N/A** |

Initial symptoms: severe leg pain for 2 months

Hospital presenting symptoms: severe leg pain for 3months (stopped meds 3 weeks ago for same problem

because developed “moon face”)

Final Diagnosis: osteoarthritis

Additional info: BP: 149/80, HR: 106, O2sat: 98, T: 36.9, RR: 19; no labs. Bought meds from medication seller

and the injection was then administered by non-Dr healthcare worker. Patient given all meds mixed

together in one packet.

Authors’ Note: Although this patient was excluded from the general calculations since she took her meds

before the two week cut-off period, she was included in the steroid numbers since one of the reasons

she came to the hospital was due to the development of “moon face”. She stopped all the meds after

noticing the change. There does not appear to be a clear indication for daily Penicillin or daily

Dexamethasone and the patient saw no improvement in her condition. Instead she developed the new

finding of “moon face” since starting NPP meds.

Abbreviations: Units (U)

NPP 82 Age: 48 Sex: F

| **Name** | **Who Prescribed** | **Dose** | **Frequency** | **Duration** | **Indication** | **Exp. date** |
| --- | --- | --- | --- | --- | --- | --- |
| **Cimetidine** | **Private clinic (non Dr)** | **200 mg** | **2qhs** | **3d** | **N/A** | **N/A** |
| **Gestid** | **Private clinic (non Dr)** | **N/A** | **N/A** | **3d** | **N/A** | **N/A** |
| **Amoxicillin** | **Private clinic (non Dr)** | **500 mg** | **q8h** | **3d** | **N/A** | **N/A** |
| **Brown pill** | **Private clinic (non Dr)** | **N/A** | **q8h** | **3d** | **N/A** | **N/A** |

Initial symptoms: palpitations, difficulty swallowing for 3 months

Hospital presenting symptoms: palpitations, difficulty swallowing for 3 months

Final Diagnosis: 1. Toxic goiter 2. Rapid atrial fibrillation

Additional info: BP: 142/76, HR: 105, T: 36.9, O2sat: 98, RR: 18; Glucose: 86, TSH: < 0.005, FT4 > 100;

Patient understands “medicine allergy”, describing itchy, red, difficult to breath and chest tightness.

States she has a known allergy. However, when asked if aware that medications can sometimes cause

serious reactions, patient said: no. When asked if the prescriber asked about past allergies, the patient

stated: no.

Authors’ Note: While the depth of the evaluation by the NPP is unknown, the true diagnosis appears to

have been missed, based on the prescribed medications, which included an antibiotic for a non-infectious

condition. Patient states the prescriber did not ask about her past medication allergy, although it is

uncertain if the patient knows the name of the offending agent.

Abbreviations: Every evening (qhs), Every 8 hours (q8h), Thyroid stimulating hormone (TSH),

Free Thyroxine (FT4

NPP 85 Age: 49 Sex: F

| **Name** | **Who Prescribed** | **Dose** | **Frequency** | **Duration** | **Indication** | **Exp. date** |
| --- | --- | --- | --- | --- | --- | --- |
| **Zyloric (Allopurinol)** | **pharmacy** | **200mg** | **2/d** | **10d** | **N/A** | **2019** |
| **Tanakan (similar to Gingko Bilboa)** | **pharmacy** | **40 mg** | **2/d** | **10d** | **N/A – for memory problems** | **6-2017** |
| **Omeprazole** | **pharmacy** | **20mg** | **2/d** | **10d** | **N/A** | **2018** |

Initial symptoms: numbness in arm for 2 months

Hospital presenting symptoms: numbness in arm for 2 months

Final Diagnosis: Right arm pain, etiology ? sciatica (radicular pain)

Additional info: BP: 115/74, HR: 75 O2 sat: 98, T: 37 RR: 20; creatinine: 37 mmol/L, H/H: 12/39,

SGOT/SGPT: 18/13, CXR and US: done. When asked if the patient understands “medication allergy,

patient answered: yes, stating she had an allergy causing ”chest tightness and uncomfortable breathing.”

However, she stated her pharmacist did not ask about allergies before prescribing above meds. When

patient was asked if medication can sometimes cause serious reactions, patient answered: no.

No past medical history of gout or hyperuricemia noted.

Authors’ Note: There does not appear to be a clear indication for Zyloric for likely radicular arm pain.

Although no serious allergic reaction occurred with above meds, pharmacist never asked about allergies

before prescribing to a patient with a likely history of medication allergy.

Abbreviations: Two per day (2/d), Ultrasound (US)

NPP 094 Age: 65 Sex: M

| **Name** | **Who Prescribed** | **Dose** | **Frequency** | **Duration** | **Indication** | **Exp. date** |
| --- | --- | --- | --- | --- | --- | --- |
| **Erythromycin** | **retail** | **N/A** | **qd** | **5d** | **N/A** | **N/A** |
| **Novalgin** | **retail** | **N/A** | **qd** | **N/A** | **N/A** | **N/A** |
| **Dexamethasone** | **retail** | **5mg** | **qd** | **N/A** | **N/A** | **N/A** |

Initial symptoms: fever, painful for 10 days

Hospital presenting symptoms: fever, painful Lt knee, swollen for 10 days

Final Diagnosis: 1. Septic arthritis (gram negative bacilli, 36,000 WBC on knee aspiration), 2. new onset DM;

HbA1C 8.4, glucose 153 mg/dl

Additional info: BP: 117/73, HR: 101, O2sat: N/A, T: 36.4, RR: 20; creatinine: 128 mmol/L,

SGOT/SGPT: 90/90, Glucose: 153 mg/dl, HbA1C: 8.4, H/H: 10/31, WBC 21,000, Blood cultures: positive

for *Burkholderia Pseudomallei.* Joint aspiration: 36,000 WBC, gram neg bacilli. When asked if new

symptoms after starting NPP medication, patient stated: worsening fever, swelling and pain in knee.

Authors’ Note: This patient presented with symptoms of septic arthritis and was prescribed Erythromycin

(not typical empiric coverage for a septic joint). Dexamethasone was also prescribed, likely worsening

his undiagnosed diabetes (a risk factor for Melioidosis, a gram negative bacterial infection commonly found

in Southeast Asia). Novalgin, a pain med, was also prescribed. This medication has reportedly been banned

in Cambodia, in addition to many other countries, due to potential serious adverse effects.

Abbreviations: milligrams per deciliter (mg/dl),

NPP 98 Age: 57 Sex: M

| **Name** | **Who Prescribed** | **Dose** | **Frequency** | **Duration** | **Indication** | **Exp. date** |
| --- | --- | --- | --- | --- | --- | --- |
| **Aureomycin** | **Retail/himself** | **500mg** | **2 tabs many times** | **N/A** | **antibiotic** | **N/A** |
| **Paracetamol** | **Retail/himself** | **500mg** | **2 tabs q8h** | **N/A** | **pain** | **N/A** |
| **Podanex (sp?) (per**  **research assistant =**  **acetaminophen)** | **Retail/himself** | **500mg** | **q8h** | **N/A** | **pain** | **N/A** |
| **Penicillin** | **Retail/himself** | **N/A** | **q12h** | **N/A** | **antibiotic** | **N/A** |
| **Ampicillin** | **Retail/himself** | **500mg** | **q12h** | **N/A** | **antibiotic** | **N/A** |
| **Prednisolone** | **Retail/himself** | **5mg** | **2 tabs q12h** | **N/A** | **N/A** | **N/A** |
| **Antacid (Maalox)** | **Retail/himself** | **N/A** | **1 tab many times** | **N/A** | **N/A** | **N/A** |

Initial symptoms: severe back pain, Lt upper abdominal pain for 10 days

Hospital presenting symptoms: severe back pain (mid back), Lt upper abdominal pain for 10 days

Final Diagnosis: 1.severe anemia due to GI bleeding 2. Acute pancreatitis?

Additional info: BP: 105/64 HR: 85 RR: 20 O2sat: 98 T: 36.6; creatinine: 61 mmol/L,

H/H 5.1/20, Serum Iron: 2.7mmol/L (nl 11-29mmol/L), SGOT/SGPT: 19/18, Glucose: 61mg/dl;

PMH: bilateral femoral head necrosis, upper GI bleed, requiring transfusion; Patient denied

any history of joint trauma, infection or childhood limp. Admitted taking steroids on and off for many

years (maybe 20 years) and states he never told anyone (family or previous doctors). About 2 years

earlier, patient developed bilateral femoral head necrosis progressing over a year before receiving

femoral head prosthetic surgery (brought in old x-rays). Bought meds on his own for years. When asked if

any new symptoms since starting NPP meds, patient stated: worse symptoms and sometimes pass stool

with blood.

Authors’ Note: This patient has no clear indication for chronic steroid usage and possibly suffered adverse

consequences. Steroid use is the most likely reason for his bilateral femoral head necrosis and also a

possible exacerbating factor in his GI bleed. (Although steroids alone may not significantly increase the

risk of GI bleed, steroids in someone with history of GI bleed may exacerbate an existing ulcer by inhibiting

healing). The patient does not have any evidence of liver abnormalities; however, the combination of two

different acetaminophen medications, taken chronically at the above prescribed doses, could be toxic.

Lastly there does not appear to be a clear indication for multiple antibiotics, two within the same class.

Abbreviations: Every 12 hours (q12h), tablets (tabs), Spelling is uncertain (sp?)

NPP 112 Age 66 Sex: F

| **Name** | **Who Prescribed** | **Dose** | **Frequency** | **Duration** | **Indication** | **Exp. Date** |
| --- | --- | --- | --- | --- | --- | --- |
| **Amlodipine** | **Retail/friend** | **5 mg** | **1-2 tabs** | **On and off** | **N/A** | **N/A** |
| **Novalgin** | **Retail/friend** | **N/A** | **q8h** | **prn** | **pain** | **N/A** |

Initial symptoms: neck tension, headache, chest tightness on and off

Hospital presenting symptoms: neck tension, headache, chest tightness on and off

Final Dx: 1. HTN, 2. Left ovarian cyst by Ultrasound, 3. anxiety

Additional info: BP: 210/85 HR: 76 RR: 26 T: 36.5 O2 sat: 98; Glucose: 78 mg/dl H/H 13.4/40,

Creatinine: 79 mmol/L, EKG: done, not found; “Patient used to use Amlodipine on and off for

more than 10 years by herself by asking somebody that had hypertension.” The friend told her to use

Amlodipine and Novalgin when she had “severe headache and neck tension”. First visit 2 weeks ago at

SHCH, BP was 230/115, new meds added this visit. PMH: HTN, thyroid disease. No NPP instructions were

given to patient since she bought meds herself based on friends advise.

Authors’ Note: Poor BP control was, in part, related to lack of understanding concerning use of BP meds.

Patient only took meds when had symptoms of “neck tension and headache”, per instructions by friend

(who also has hypertension). After patient was initially treated at SHCH hospital, a follow up appointment

was scheduled within 2 weeks to assess medication response. This is something that may or may not be

recommended by NPP sellers. Lastly, patient bought Novalgin, which is reportedly banned in Cambodia and

many other countries, due to potential serious side effects.

Abbreviations: As needed (prn), Electrocardiogram (EKG

NPP 126 Age: 58 Sex: F

| **Name** | **Who Prescribed** | **Dose** | **Frequency** | **Duration** | **Indication** | **Exp. Date** |
| --- | --- | --- | --- | --- | --- | --- |
| **Ampicillin** | **Pharmacy/self** | **500 mg** | **qd** | **3d** | **N/A** | **N/A** |
| **Vitamins** | **Pharmacy/self** | **N/A** | **N/A** | **N/A** | **N/A** | **N/A** |
| **Unknown Injection IM** | **Health center (nurse)** | **N/A** | **N/A** | **N/A** | **N/A** | **N/A** |
| **Unknown IVF** | **Health center (nurse)** | **N/A** | **N/A** | **N/A** | **N/A** | **N/A** |

Initial symptoms: About 3 weeks ago patient had consulted with doctor and was given home meds for 10

days for cough. Not better, so went to pharmacy to buy Ampicillin and vitamins herself. One week ago,

after buying pharmacy meds, she developed dizziness and vomiting so called health center and nurse

gave IV fluids and an injection, then she felt better.

Hospital presenting symptoms: cough for 1 month, sore throat

Final Diagnosis: 1. Gastritis, 2. Viral cold, 3. Anxiety, 4. Elevated lipids

Additional info: BP: 136/86 HR: 94 RR: 20 O2sat: 96 Temp: 37.5; Patient states she knew the name of a

medication (Ampicillin) from “people that live near her home who told her those who took this

medication got better”, so she went to the pharmacy to buy it herself. Advised by the pharmacist that

if she does not take the medication “regular with time and date”, there was an increased risk of drug

resistance. Patient had been a previous medication seller, stopped 2 years ago. Patient knows about

medication allergies since she had a skin rash after taking Penicillin. When asked if aware that

medication can sometimes cause serious reactions, responded: ”afraid, but still getting injections

because still sick”.

Authors’ Note: Patient stated she had an allergy to Penicillin, describing a skin rash, yet inappropriately

prescribed for herself Ampicillin because “people that live near her home told her those who took this

medication got better”. Uncertain if the dizziness and vomiting were possible allergic reaction/side

effect to the Ampicillin since these symptoms occurred soon after taking the medicine and required

her to go to the health center for IVF and an injection. Not enough information/timing to determine

if true allergic reaction. However, either way, there was there was a lack of recognition of potential

risk for repeat allergic reaction, when taking another medicine within the same class. This was

someone who had previously advised and sold medicines to others. It is noteworthy that the

pharmacist advised about the potential risk of drug resistance from inconsistent use of the antibiotics.

Abbreviations: Intramuscular (IM), Intravenous fluids (IVF)

NPP 138 Age: 35 Sex: F

| **Name** | **Who Prescribed** | **Dose** | **Frequency** | **Duration** | **Indication** | **Exp. Date** |
| --- | --- | --- | --- | --- | --- | --- |
| **Unknown** | **Health center** | **N/A** | **3x/d** | **2m on and off** | **Severe pain** | **N/A** |
| **Unknown** | **Health center** | **N/A** | **3x/d** | **2m on and off** | **Severe pain** | **N/A** |
| **Unknown** | **Health center** | **N/A** | **3x/d** | **2m on and off** | **Severe pain** | **N/A** |
| **Unknown** | **retail** | **N/A** | **2x/d** | **7m on and off** | **Severe pain** | **N/A** |
| **Unknown** | **retail** | **N/A** | **2x/d** | **7m on and off** | **Severe pain** | **N/A** |
| **Unknown** | **retail** | **N/A** | **2x/d** | **7m on and off** | **Severe pain** | **N/A** |
| **Unknown** | **retail** | **N/A** | **2x/d** | **7m on and off** | **Severe pain** | **N/A** |
| **Unknown** | **retail** | **N/A** | **2x/d** | **7m on and off** | **Severe pain** | **N/A** |
| **Unknown** | **retail** | **N/A** | **2x/d** | **7m on and off** | **Severe pain** | **N/A** |

Initial symptoms: body painful, loss of hair for 1 week

Hospital presenting symptoms: body painful especially on right side on and off for 1 year

Final Diagnosis: 1. L5-S1 narrowing of vertebral/disk space

Additional info: BP: 130/86 HR: 103 RR: 20 O2sat: 98 Temp: 36.6; X-ray spine: possible compression

fracture, L5,S1 narrowing; Patient went to a medication salesman first, then after 5 months went to

health clinic where they recommended stop the medication seller meds. But patient did not follow

instructions and still used on and off. When asked if she had any new symptoms after starting the

NPP medications the patient stated: “moon face and body is still painful”, although the medication

made her better for a while. Patient also states she has a history of “drug allergy” but does not

know the names of any of the 9 medications she is taking, nor does she know the symptoms of

allergies or that medication can cause serious reactions. Patient sates she was never given any

information concerning the side effects of the meds.

Authors’ Note: Although the medication names are unknown, patient stated a new symptom since starting

medication was “moon face”, making it highly possible that at least one (or more) med was a steroid.

However, there does not seem to be an indication for chronic oral steroids for “body painful”. The

patient told her prescribers she had an drug allergy, yet she is unaware of the any of the names of her

present medications. It is also noteworthy that the health center advised the patient to stop all outside

medication while using the clinics meds (the local hospital does this too), yet the patient continued the

both meds, which may result in unknowingly doubling the dose of commonly prescribed pain meds,

such as acetaminophen .

Abbreviations: Lumbar (L), Sacral (S), twice per day (2x/d), three times per day (3x/d)

NPP 277 Age: 60 Sex: M

| **NPP Medicine** | **Who Prescribed** | **Dose** | **Frequency** | **Duration** | **Indication** | **Exp. Date** |
| --- | --- | --- | --- | --- | --- | --- |
| **Prednisolone** | **retail** | **5 mg** | **q12h** | **3y on and off**  **Stopped 2y ago** | **Skin problem** | **N/A** |
| **Glibenclamide** | **retail** | **5 mg** | **qd** | **On and off**  **x 5y** | **N/A** | **N/A** |
| **Chlorpheniramine** | **retail** | **10 mg** | **qd** | **On and off**  **x 5y** | **N/A** | **N/A** |
| **Metformin** | **retail** | **850mg** | **qd** | **On and off**  **x 5y** | **N/A** | **N/A** |
| **Kenacort injection** | **N/A** | **N/A** | **N/A** | **10yrs but none in past 2 weeks** | **Skin problem** | **N/A** |

Initial symptoms: blurring of vision, frequent urination and weakness x 4m – [occurred 2 y ago]

(Went to an MD who only took fasting blood sugar, about 2 years ago: 360 mg/dl)

Hospital presenting symptoms: abdominal pain radiating to back, feeling burning both legs for 1 month

Final Diagnosis: 1. DM II, peripheral neuropathy, HbA1C: 5.8, 2. HTN, 3. Psoriasis

Additional info: BP: 163/91, HR: 82, T: 36.1, RR:20, O2sat: 99; Creatinine: 69mmol/L, HbA1C: 5.8

Note: Patient has received NPP IM Kenacort injections for skin problems for 10 years, still using but not

within the past 2 weeks. PMH: DM, HTN, skin problem. Patient was never given any info concerning side

effects of the meds he was taking and is completely unaware that medicines can cause serious problems.

Hospital doctor stopped his Glibendclamide.

Authors’ Note: This patient had been taking both steroids and his DM meds for the past 5 years on and off.

His HbA1C is now in a borderline normal range despite his irregular usage of the DM meds. Although the

HbA1C could be a lab error, more likely his sugars were elevated in the past because of the double

steroid usage (oral for 3 years concurrent with IM steroids for past 10 years) and have now returned to a

more normal level because he stopped oral steroids 2 years ago. The hospital doctor continued the

Metformin, but stopped the Glibenaclamide; perhaps no need for any DM meds, if chronic steroids are

stopped. If patient has a true peripheral neuropathy, which doctor attributed to his DM, then this may

have been avoided if steroids had been used judiciously.

Abbreviations: Year (y)

NPP 302 Age: 63 Sex: F

| **NPP Medicine** | **Who Prescribed** | **Dose** | **Frequency** | **Duration** | **Indication** | **Exp. Date** |
| --- | --- | --- | --- | --- | --- | --- |
| **Unknown** | **retail** | **N/A** | **2tab q12h** | **3d** | **Fever and chills** | **N/A** |
| **Unknown** | **retail** | **N/A** | **2tab q12h** | **3d** | **Fever and chills** | **N/A** |
| **Unknown** | **retail** | **N/A** | **2tab q12h** | **3d** | **Fever and chills** | **N/A** |
| **Unknown IM inj** | **Private clinic – non-Dr** | **N/A** | **N/A** | **N/A** | **Fever and chills** | **N/A** |
| **Unknown IM inj** | **Private clinic – non-Dr** | **N/A** | **N/A** | **N/A** | **Fever and chills** | **N/A** |
| **Unknown IM inj** | **Private clinic – non-Dr** | **N/A** | **N/A** | **N/A** | **Fever and chills** | **N/A** |

Initial symptoms: Headache, vomiting, dizziness for 3 days

Hospital presenting symptoms: vomit, headache, dizziness for half a month

Final Diagnosis: 1. Viral meningitis, 2. Spinal compression fracture

Additional info: BP: 124/77, HR: 82, O2st: 97, T: 37.8 (axillary), RR: 19; creatinine: 78 mmol/L,

SGOT/SGPT: 23/20, H/H: 9.8/31 MCV: 79, HbA1C: 6.03, CXR done, osteoporotic; T12, L2,3,5;

Lumbar Puncture: WBC: 83; PMH: back pain and GERD; Has received injections in the past

from private clinic, non-doctor. Patient admitted to the hospital and treated with antibiotics.

Authors’ Note: The names of the medications and injections are unknown. Given the symptoms, likely one

or more antibiotic was prescribed, however; there is no way hospital doctors can determine if this

occurred or if the type or dosing was appropriate. While the admitting diagnosis was “Viral

Meningitis”, antibiotics were still started because clinical picture and cultures/cell counts may be altered

after partial antibiotic treatment. (This patient still has a low grade fever/symptoms after 2 weeks of

retail med and injection therapy). Because past treatment is unknown, likelihood of using prolonged,

repetitive and/or potentially unnecessary antibiotic coverage at the hospital is increased, while the true

diagnosis becomes muddled in this region of the world where the differential diagnosis is broad, i.e. viral,

bacterial, TB, Leptospirosis, scrub typhus, etc. However, if this was a true bacterial infection, then an

early start with an appropriate antibiotic may be lifesaving and might not be readily available
 without the presence of NPPs.

Abbreviations: Thoracic vertebra 12 (T 12), Lumbar vertebrae 2,3,5 (L 2,3,5), Gastroesophageal Reflux

Disease (GERD)

NPP 313 Age: 63 Sex: F

| **NPP Medicine** | **Who Prescribed** | **Dose** | **Frequency** | **Duration** | **Indication** | **Exp. Date** |
| --- | --- | --- | --- | --- | --- | --- |
| **Diamicron** | **Retail (although initial dx at health center, didn’t follow up and retail didn’t check sugar)** | **30mg** | **qd** | **10m** | **DM II** | **N/A** |
| **paracetamol** | **Retail** | **500mg** | **q8h** | **prn** | **Fever** | **N/A** |
| **Unknown** | **Retail** | **N/A** | **N/A** | **On and off** | **Leg numbness** | **N/A** |
| **Unknown** | **Retail** | **N/A** | **N/A** | **On and off** | **Poor sleep** | **N/A** |

Initial symptoms: N/A, however, patient initially went to a health center and was diagnosed with DM by

blood test. Did not follow up but instead went to retail/med seller near home for meds, which were given

without checking blood sugar.

Hospital presenting symptoms: body weakness, both leg chronic wound for 3 months

Final Diagnosis: 1. DM II, 2. Peripheral neuropathy, 3. HTN, 4. First, 2^nd^ and 3^rd^ toe wounds, 5. Renal

insufficiency, creatinine clearance: 45 ml/min

.

Additional info: BP: 180/92, HR: 78, O2 sat: 96, T: 36.3, RR: 20; creatinine: 88 mmol/L, SGOT/SGPT: 24/30,

glucose: 362mg/dl, HbA1C: 12.23, H/H: 10/31; Understands allergic reactions and has had “sweating,

chest tightness, redness and face swollen” after using Ampicillin, Penicillin, and “Clamoxicillin” (used for

vaginal discharge and irritation). Patient has received NPP injections in the past.

Authors’ Note: Although initial diagnosis was made at the health center, patient chose to buy meds from a

retail seller and received no follow up. Now has renal insufficiency, foot ulcers and poorly controlled

DM, in addition to undiagnosed hypertension. Interestingly, although no diagnosis or prescriber

information is available for her past vaginal symptoms, it is possible this patient was inadvertently

(and likely inappropriately) prescribed antibiotics multiple times for a yeast infection (common in poorly

controlled DM) because it was unrecognized that all the antibiotics chosen were from the same class of

medication, Penicillins, to which she is allergic.

Abbreviations: Milliliter per minute (ml/min)

NPP 442 Age: 51 Sex: M

| **NPP Medicine** | **Who Prescribed** | **Dose** | **Frequency** | **Duration** | **Indication** | **Exp. Date** |
| --- | --- | --- | --- | --- | --- | --- |
| **Dexamethasone** | **Retail** | **0.5mg** | **2tab** | **PRN on and off**  **x 8y** | **pain** | **N/A** |
| **Piroxicam** | **Retail** | **20mg** | **1tab** | **PRN on and off**  **x 8y** | **pain** | **N/A** |
| **Analgen** | **Retail** | **500mg** | **1tab** | **PRN on and off**  **x 8y** | **pain** | **N/A** |
| **MTV** | **Retail** | **NA** | **1tab** | **PRN on and off**  **x 8y** | **pain** | **N/A** |
| **Amoxicillin** | **Retail** | **500mg** | **1tab** | **PRN on and off**  **x 8y** | **pain** | **N/A** |
| **Khmer traditional**  **with wine** | **N/A** | **N/A** | **N/A** | **PRN on and off**  **x 8y** | **pain** | **N/A** |
| **Unknown injection**  **Intra-articular (knee)** | **N/A (non-Dr)** | **N/A** | **5 times in the past** | **N/A** | **Pain and fever** | **N/A** |

Initial symptoms: multiple join pain, fever and chills for 3 days

Hospital presenting symptoms: multiple joint pain and deformities for 8 years

Final Diagnosis: 1. Osteoarthritis both knees, 2 alcoholism, 3. Renal insufficiency (GFR: 51.9), 4. Pseudogout

[Discharged with prescriptions for Allopurinol, Colchicine and Paracetamol.]

Additional info: BP: 150/90, HR: 97, O2 sat: 98, T: 37, RR: 20; creatinine: 112 mmol/L (hi),

HbA1C: 6.4, H/H: 12.7/NA, SGOT/SGPT: 39/72, uric acid: 667micromol/L (hi > 448). Per chart, patient

“now [has] appearance looking moon face”. Had NPP injections both knees 5 times in the past.

Authors’ Note: Although uncertain of true diagnosis based just on labs and symptoms, (no X-Ray or joint

fluid results available on chart), there does not appear to be an infectious indication for the antibiotic

which was reportedly used for “pain”, on and off for 8 years. Although steroids may be beneficial in short

courses for many types of arthritis, other treatment would likely be a better choice to avoid chronic use

side effects. Patient noted to have a “moon face”, elevated HbA1C and elevated BP, all which may

be related to 8 years of frequent steroid usage. Analgen is reportedly banned in Cambodia

(and other countries) due to potential serious adverse effects. Lastly, the patient reports receiving

multiple past unknown intra-articular injections from a non-physician.

NPP 472 Age: 61 Sex: F

| **NPP Medicine** | **Who Prescribed** | **Dose** | **Frequency** | **Duration** | **Indication** | **Exp. Date** |
| --- | --- | --- | --- | --- | --- | --- |
| **Diamicron** | **Private clinic**  **unknown if Dr** | **30mg** | **1½ tab q12h** | **5y** | **DM II** | **N/A** |
| **Glibenclamide** | **Private clinic**  **unknown if Dr** | **5mg** | **q12h** | **5y** | **DM II (when**  **uncontrolled)** | **N/A** |
| **Unknown** | **Pharm/retail** | **N/A** | **2x/d** | **2m** | **Cough and SOB** | **N/A** |
| **Unknown** | **Pharm/retail** | **N/A** | **2x/d** | **2m** | **Cough and SOB** | **N/A** |
| **Unknown** | **Pharm/retail** | **N/A** | **2x/d** | **2m** | **Cough and SOB** | **N/A** |
| **Unknown** | **Pharm/retail** | **N/A** | **2x/d** | **2m** | **Cough and SOB** | **N/A** |
| **Unknown** | **Pharm/retail** | **N/A** | **2x/d** | **2m** | **Cough and SOB** | **N/A** |
| **Unknown Injection**  **IM (prior to 2 w)** | **Pharm/retail** | **N/A** | **N/A** | **N/A** | **Cough and SOB** | **N/A** |
| **Unknown Injection**  **IVF (prior to 2 w)** | **Pharm/retail** | **N/A** | **N/A** | **N/A** | **Cough and SOB** | **N/A** |

Initial symptoms: cough with sputum for 2 months, sweating, weakness

Hospital presenting symptoms: sweating, tremor, cough with sputum for 2 months, weakness

Final Diagnosis: 1. Sepsis/Military TB, 2. Microcytic Anemia (H/H: 9.9/29 MCV: 74), 3. DM II – (HbA1C: 7.37);

Admitted. TB meds started by TB clinic doctors at SHCH.

Additional info: BP:122/84, HR: 146, O2 sat: N/A, T: 38.1, RR: 26; creatinine: 100 mmol/L WBC: 15,070,

HbA1C: 7.32, SGOT/SGPT: 21/12, H/H: 9.9/29; US: done. The IVF, IM injections were prescribed prior

to 2 weeks but for the same cough and SOB. TB review committee at the hospital (SHCH) started TB meds.

Authors’ Note: Although uncertain what meds were prescribed to patient for her cough and SOB, no

improvement occurred despite 2 months of therapy. TB meds should not typically be sold from a

pharmacy/retail store. ^33^ In Phnom Penh, pharmacy owners have been educated to refer to TB clinic. This

patient was not from Phnom Penh. However, if the unknown meds were actually TB meds, then it is

unlikely diagnostic tests were performed to confirm diagnosis or evaluate for resistance. Monitoring the

patient and assessing close contacts for disease would also be unlikely, increasing the chances for disease

spread. Conversely, if the meds were not TB meds, then, in addition to the delay in receiving

appropriate therapy, limited resources were used to buy unneeded meds/injections for 2 months.

Abbreviations: Shortness of breath (SOB)
